# p62/SQSTM1 accumulation due to degradation inhibition and transcriptional activation plays a critical role in silica nanoparticle-induced airway inflammation via NF-κB activation

**DOI:** 10.1186/s12951-020-00634-1

**Published:** 2020-05-19

**Authors:** Yifan Wu, Yang Jin, Tianyu Sun, Piaoyu Zhu, Jinlong Li, Qinglin Zhang, Xiaoke Wang, Junkang Jiang, Gang Chen, Xinyuan Zhao

**Affiliations:** 1 Department of Occupational Medicine and Environmental Toxicology, School of Public Health, Nangtong University, Nantong, 226019 China; 2 School of Pharmacy, Nantong University, Nantong, 226001 China; 3 Departments of Gastroenterology, Affiliated to Wuxi People’s Hospital, Nanjing Medical University, Wuxi, 214023 China

**Keywords:** Silica nanoparticle, Airway inflammation, p62 accumulation, Autophagic flux blockade, Transcriptional activation, Nrf2

## Abstract

**Background:**

Most nanoparticles (NPs) reportedly block autophagic flux, thereby upregulating p62/SQSTM1 through degradation inhibition. p62 also acts as a multifunctional scaffold protein with multiple domains, and is involved in various cellular processes. However, the autophagy substrate-independent role of p62 and its regulation at the transcriptional level upon NPs exposure remain unclear.

**Results:**

In this work, we exposed BEAS-2b cells and mice to silica nanoparticles (SiNPs), and found that SiNPs increased p62 protein levels in vivo and vitro. Then, we further explored the role and mechanism of SiNPs-stimulated p62 in vitro, and found that p62 degradation was inhibited due to autophagic flux blockade. Mechanistically, SiNPs blocked autophagic flux through impairment of lysosomal capacity rather than defective autophagosome fusion with lysosomes. Moreover, SiNPs stimulated translocation of NF-E2-related factor 2 (Nrf2) to the nucleus from the cytoplasm, which upregulated p62 transcriptional activation through direct binding of Nrf2 to the p62 promoter. Nrf2 siRNA dramatically reduced both the mRNA and protein levels of p62. These two mechanisms led to p62 protein accumulation, thus increasing *interleukin* (*IL*)-*1* and *IL*-*6* expression. SiNPs activated nuclear factor kappa B (NF-κB), and this effect could be alleviated by p62 knockdown.

**Conclusion:**

SiNPs caused accumulation of p62 through both pre- and post-translational mechanisms, resulting in airway inflammation. These findings improve our understanding of SiNP-induced pulmonary damage and the molecular targets available to mitigate it.

## Background

Engineered nanoparticles (NPs), with diameters less than 100 nm, are widely used in several fields, including industry and medicine [1]. Among NPs, silica nanoparticles (SiNPs) are one of the most commonly applied types worldwide [2]. Recently, public concern has been raised about their harmful effects on human health and the environment. The respiratory system is thought to be an important pathway through which NPs can access the human body [3]. Once inhaled, SiNPs can lead to pulmonary inflammation, apoptosis of alveolar epithelial cells (AECs), and ultimately pulmonary fibrosis [4, 5]. Despite intense examination, SiNP-associated physiological impacts on the pulmonary system and their underlying molecular mechanisms remain largely unclear.

Autophagy is a ‘self-eating’ process involving the engulfing of organelles, molecular complexes, and cytoplasmic proteins into autophagosomes [6]. After initiation, the complete autophagic process involves fusion of autophagosomes with lysosomes and subsequent cargo degradation. This dynamic process of autophagosome formation, subsequent fusion with a lysosome, and finally degradation of its contents is referred to as autophagic flux. Appropriate autophagic flux is critical to human health, and its dysfunction may be related to various pulmonary pathologies, including inflammation and AEC apoptosis [7, 8]. Interestingly, nearly all NPs aside from SiNPs cause abnormal cellular outcomes through autophagy dysfunction [9]. Notably, SiNP-mediated autophagy dysfunction may be due to excessive induction of autophagy and/or blockade of its flux, leading to cell death and other pathologies [5, 10]. The p62 protein, also called sequestosome 1, is another common marker of the autophagy process, along with microtubule-associated protein 1 light chain-3 protein (LC3). This protein functions as a critical receptor that identifies and binds to ubiquitinated proteins, delivering them via membrane-bound LC3 to the phagophore for subsequent degradation. Meanwhile, p62 itself is degraded by autolysosomes following the initiation of autophagy. Thus, p62 accumulation has been identified as a general marker of reduced autophagic flux [11].

In addition to providing a substrate for autophagy, p62 also acts as a multifunctional scaffold protein that contains multiple domains and is reportedly involved in a variety of cellular processes through interactions with different binding partners. It can serve as a signalling hub for multiple cellular signal transduction cascades related to cell survival, cell death, inflammation, and oxidative stress [12]. For example, p62 promotes aggregation of cullin3 (CUL3)-modified caspase-8 within p62-dependent foci, leading to full activation and cell death [13]. Moreover, p62 plays an important role in HAMLET-induced apoptotic cell death through caspase-8 activation [14]. Most studies of nanotoxicology have measured p62 protein levels to assess autophagic flux, and several NPs have been found to cause p62 accumulation through defect of lysosome-autophagosome fusion and lysosome dysfunction [5, 15]. However, those studies ignored the autophagy substrate-independent role of p62 under NPs exposure. Recently, Feng et al. reported that graphene oxide (GO) induced aberrant p62 accumulation through impairment of lysosome degradation and subsequent blockade of autophagic flux, leading to eventual caspase-mediated apoptosis. Moreover, p62 knockdown led to downregulation of cleaved-caspase 3, cleaved-caspase 9, and Bax expression, supporting GO-triggered p62 accumulation as a pro-apoptosis mechanism [16]. When treated with certain environmental stimuli, p62 was regulated in a pre-translation manner without autophagic flux blockade. Our group previously found that antimony trichloride enhanced autophagic flux and upregulated p62 protein expression in A549 cells [17].

In the present study, we systematically investigated the molecular mechanisms and biological role underlying SiNP-mediated p62 regulation. We observed that both impairment of autophagic flux and transcriptional activation contributed to SiNP-induced p62 accumulation, leading to nuclear factor kappa B (NF-κB) activation and airway inflammation. These findings provide confirmation of the detailed mechanism of p62 accumulation in SiNP-associated pulmonary toxicity.

## Materials and methods

### Reagents, antibody and plasmids

The following reagents: cycloheximide (CHX) (C4859), chloroquine (CQ) (C6686), 3-methyladenine (3-MA) (M9281), and epidermal growth factor (EGF) (E9644), actinomycin D (A4262) are obtained from Sigma (Missouri, USA). The SiNPs were gifts from Dr. Zhenfeng Zhu (State Key Laboratory of Modern Optical Instrumentation; Zhejiang University). Their synthesis was described in our previous study [18]. The primary antibodies include anti-LC3 (Rabbit, L7543, Sigma, Missouri, USA), anti-p62 (Rabbit, P0067, Sigma, Missouri, USA), anti-ACTB (Mouse, A5316, Sigma, Missouri, USA), anti- epidermal growth factor receptor (EGFR, Rabbit, 4267, Cell signaling, Massachusetts, USA), anti-NF-κB (Rabbit, ab16502, Abcam, Cambridge, UK), anti-p-NF-κB (Rabbit, ab86299, Abcam, Cambridge, UK), anti- NF-E2-related factor 2 (Nrf2, Rabbit, 16396-1-AP, Proteintech, Chicago, USA), anti-Lamin A/C (Rabbit, 10298-1-AP, Proteintech, Chicago, USA), anti-Tubulin (Rabbit, 10068-1-AP, Proteintech, Chicago, USA), anti-acetylated histone H3 (Lys9/18) (Ac-H3, K9/18, Rabbit, 07-593, Millipore, massachusetts, USA), anti-K4-dimethylated histone H3 (H3K4me2, Rabbit, 49-1004, Invitrogen, California, USA). The secondary antibodies include HRP-conjugated Goat anti-Rabbit secondary antibody (RS0002, Immunoway, State of Texas, USA) and HRP-conjugated Goat anti-Mouse secondary antibody (RS0001, Immunoway, State of Texas, USA). The plasmids comprising the genes of green fluorescent protein (GFP)-tagged LC3 (GFP-LC3), yellow fluorescent protein (YFP)-tagged LAMP1 (YFP-LAMP1) and cyan fluorescent protein (CFP)-tagged LC3 (CFP-LC3) were kindly provided by Prof. Wei Liu (Department of Biochemistry and Molecular Biology; Zhejiang University School of medicine). The pGL3.0 plasmid was provided by Prof. Xiangwei Gao (Institute of Environmental Medicine, Zhejiang University School of medicine) [3].

### Cell culture, transfection, autophagy induction and SiNPs treatment

The human bronchial epithelial cells (BEAS-2b) cells were purchased from Shanghai Cell Bank, and cultured in Dulbecco’s Modified Eagle Medium/Nutrient Mixture F-12 (DMEM/F-12) supplemented with 10% fetal bovine serum (FBS, Gibco, New York, USA) at 37 °C in a humidified atmosphere with 95% air and 5% CO_2_. The transient transfection of the plasmids (GFP-LC3, YFP-LAMP1 and CFP-LC3) were performed using DNA Transfection Reagent (11668-019, Invitrogen, California, USA) according to manufacturer’s instruction. The cells were harvested 48 h after transfection. For other experiments, the transfected cells were replated and incubated for another 12 h followed by indicated SiNPs exposure.

To downregulate p62 or Nrf2, BEAS-2b cells were transiently transfected with 100 pmol of the chemically synthesized siRNAs targeting Nrf2, p62 or the negative control siRNA using LipofectamineRNAiMAX (Invitrogen, California, USA) following the manufacturer’s protocol. siRNAs were synthesized by GenePhama company (Shanghai, China) (siNrf2, sense: 5′-GAAUGGUCCUAAAACACCATT-3′, anti-sense: 5′-UGGUGUUUUAGGACCAUUCTG-3′; sip62, sense: 5′-GUGACGAGGAAUUGACAAUTT-3′, anti-sense: 5′-AUUGUCAAUUCCUCGUCACTT-3′; siNC, sense: 5′-UUCUCCGAACGUGUCACGUTT-3′, anti-sense: 5′-ACGUGACACGUUCGGAGAATT-3′).

For autophagy induction, the BEAS-2b cells were treated with starvation medium (1% bovine serum albumin (BSA), 1 mM CaCl_2_, 140 mM NaCl, 1 mM MgCl_2_, 20 mM HEPES, and 5 mM glucose, pH 7.4) at 37 °C for 2 h.

For SiNPs exposure experiments, BEAS-2b cells were incubated with DMEM/F-12 supplemented with 10% FBS for 24 h. Before treatment, SiNPs were sonicated at 200 W for 1 min prior to cell or mouse treatment. Then, BEAS-2b cells were exposed to SiNPs suspended in DMEM/F-12 supplemented with 1% FBS at indicated conditions. The doses used in present study were 0, 0.625, 12.5, 25, 50 μg/mL can lead to cytotoxicity in a dose-dependent manner as we reported previously [19].

### p62 mRNA and protein degradation assay

After protein synthesis inhibition by cycloheximide, the p62 protein levels were affected by degradation. Therefore, for p62 protein degradation analysis, BEAS-2b cells treated with 10 μM cycloheximide for 1 h were exposed to SiNPs at 50 μg/mL for 3, 6, 9 h, followed by detection of p62 protein levels using immunoblotting analysis.

For *p62* mRNA degradation analysis, the BEAS-2b cells pretreated with 2 μg/mL actinomycin D (RNA synthesis inhibitor) were treated with or without SiNPs at 50 μg/mL for 3, 6, or 9 h, and the *p62* mRNA level was measured by Quantitative real-time polymerase chain reaction (qRT-PCR).

### Autophagosome degradation assay

BEAS-2b cells were treated with SiNPs at 50 μg/mL for 12 h or starvation medium for 2 h, followed by treatment with 10 mM of 3-MA to suppress new autophagosomes synthesis. Once synthesis of autophagosomes were inhibited, the autophagosomes were gradually degraded. The LC3 expression levels were detected by Western blot at 20, 40, 60 min later. Also, BEAS-2b cells transiently expressing GFP-LC3 were treated as mentioned above, then GFP signal was imaged by confocal microscopy.

### qRT-PCR

Total RNA was isolated with Trizol reagent (Invitrogen, California, USA) according to the manufacturer’s protocol. We performed qRT-PCR assay as described before [17]. In brief, real-time quantitative PCR was performed in SYBR GREEN PCR Master Mix (Applied Biosystems, Waltham, USA) in 10 μL reactions. The primers used are described in Additional file 1: Table S1. The related mRNA expression levels of *p62*, *Nrf2*, *interleukin* (*IL*)-*1* as well as *IL*-*6* were normalized to the *β*-*actin*.

### Immunoblotting analysis

After sample collection, total proteins were extracted and quantified using bicinchonininc acid protein assay kit (Beyotime, P0009, Shanghai, China). The equal proteins were resolved by sodium dodecyl sulfate polyacrylamide gel electrophoresis and transferred to NC membrane. The membrane was blocked by 3% BSA contained in Tris Buffered saline Tween (TBST, 20 mM Tris–HCl, pH 8.0; 150 mM NaCl; 0.05% Tween-20) buffer for 2 h at room temperature, followed by incubation with corresponding primary antibody at 4 °C overnight. Then, the membranes were incubated with corresponding secondary antibodies for 1 h. Finally, the protein bands were imaged using an enhanced chemiluminescence system (ECL; Millipore, P90720, massachusetts, USA). Image J was applied to calculate protein levels.

### Immunofluorescent staining

The coverslips for cell culture were washed with phosphate buffered saline (PBS) for 3 times and fixed with 4% paraformaldehyde for 15 min at 4 °C. Then, the cells were permeabilized with 0.25% Triton X-100 for another 15 min at 4 °C. The goat serum (Zhongshan Golden Bridge Biotechnology, #ZLI-9021, Beijing, China) was used to block non specific binding sites for 2 h at room temperature. The cells were further incubated with specific primary antibody overnight at 4 °C, followed by incubation with corresponding secondary antibody for 1 h. 4′,6-diamidino-2-phenylindole (DAPI) incubation for 10 min was used to mark nuclei. Finally, the coverslips were transferred to glass slide, and mounted with 87% glycerol. The cells were visualized with a fluorescence microscope (Leica, DM4000B, Munich, Germany).

### Prepare of nuclear and cytoplasmic proteins

After exposure, the cells were collected. We performed cytoplasmic and nuclear fractions separation using nuclear and cytoplasmic protein extraction kit (Beyotime, P0027, Shanghai, China). Briefly, cells were resuspended in PBS in addition to cytoplasmic protein extraction reagent A and B. Then, the samples were centrifuged, and the supernatant was collected as cytoplasmic fraction. The nuclei were resuspended in nuclear protein extraction agent. The supernatant was collected as the nuclear fraction after centrifugation.

### Chromatin immunoprecipitation (ChIP)

In brief, The DNA fragments of BEAS-2b treated as indicated were precleared with salmon sperm DNA/protein A-agarose to remove unspecific binding. Then, the lysates were incubated with 2 μg of anti-acetylated histone H3, anti-K4-dimethylated histone H3, anti-Nrf2 or control IgGs at 4 °C overnight, followed by extraction of immunoprecipitated DNA. The purified DNA was amplified by real time PCR with SYBR GREEN PCR Master Mix. Sequences of the primers used for p62 promoter amplification are listed in Additional file 1: Table S1.

### p62 promoter plasmid construction and luciferase assay

For the p62 promoter plasmid construction, p62 promoter region containing 1000 bp fragment was amplified by PCR from human genomic DNA using the forward and reverse primers. Then, the PCR production was cloned to pGL3.0 control plasmid between XhoI and HindIII sites. BEAS-2b cells were plated in 35-mm dish 24 h before transfection, followed by transfection of p62 promoter bearing plasmid. pRL-TK was always co-transfected as the internal control. After transfection for 24 h, BEAS-2b cells were exposed to SiNPs for another 12 h. Then, the cells were harvested, lysed and centrifuged for further luciferase activity analysis using Dual-Luciferase analysis system (Promega, Wisconsin, USA) according to the manufacturer’s instructions. The firefly luciferase activation was normalized to renilla luciferase.

### EGFR degradation assay

The BEAS-2b cells were treated with 50 μg/mL SiNPs for 12 h, then were exposed to 10 μM CHX for another 4 h. Then, exposure to 100 ng/mL EGF for 15 min stimulated EGFR accumulation. After EGF binds to its receptors, the receptor complex undergoes endocytosis and is targeted to lysosomes for degradation. Then EGFR protein expression levels were detected by Western blot or immunofluorescent assay to indicate lysosomal degradation capacity.

### Animal treatment

All animal experiments were conducted according to the ethical guidelines by the Ethics Committee of Laboratory Animal Care and Welfare, Nantong University. (ICR) male mice at 6 weeks were anesthetized with isoflurane using the isoflurane dispenser (Drager, catalog number: 16-7001, Lubeck, Germany), instilled with SiNPs at the concentration of 5.0 mg/kg for three times per week [19]. One week later, biopsies of lung tissues were fixed with 10% paraformaldehyde in sterile phosphate-buffered saline, embedded in paraffin wax, and sectioned at 5 μm for further p62 staining. On the other hand, p62 expression levels were also detected by Western blot.

### Immunohistochemistry studies

The lung tissue slice with 5 μm thickness was subjected to further immunohistochemistry assay. The sections were deparaffinized and rehydrated, and boiled in 10 mM citrate buffer for 30 min for antigen retrieval. Endogenous peroxidase was blocked for 10 min using 3% dihydrogen dioxide. Then, the slides were blocked with 3% BSA at room temperature, followed by incubation with p62 antibody at 4 °C overnight. Then, we used corresponding secondary antibody to incubate slides for 1 h, and visualized with 3,3-diaminobenzidine substrate (Zhongshan Golden Bridge Biotechnology, #PV-9000-D, Beijing, China). Finally, the sections were counterstained with hematoxylin.

### Statistical analysis

Comparison between two groups (Control and SiNPs) were performed with Student’s two-tailed t test. One-way ANOVA was used to determine statical significance for multiple groups analysis. All statistical analyses were calculated using GraphPad Prism version 6.0. A *p* value ≤ 0.05 was considered significant. Differences were considered statistically significant if *p *< 0.05 (*), *p *< 0.01 (**), or not significant (NS).

## Results

### SiNPs induced p62 accumulation in vitro and in vivo

**Fig. 1:**
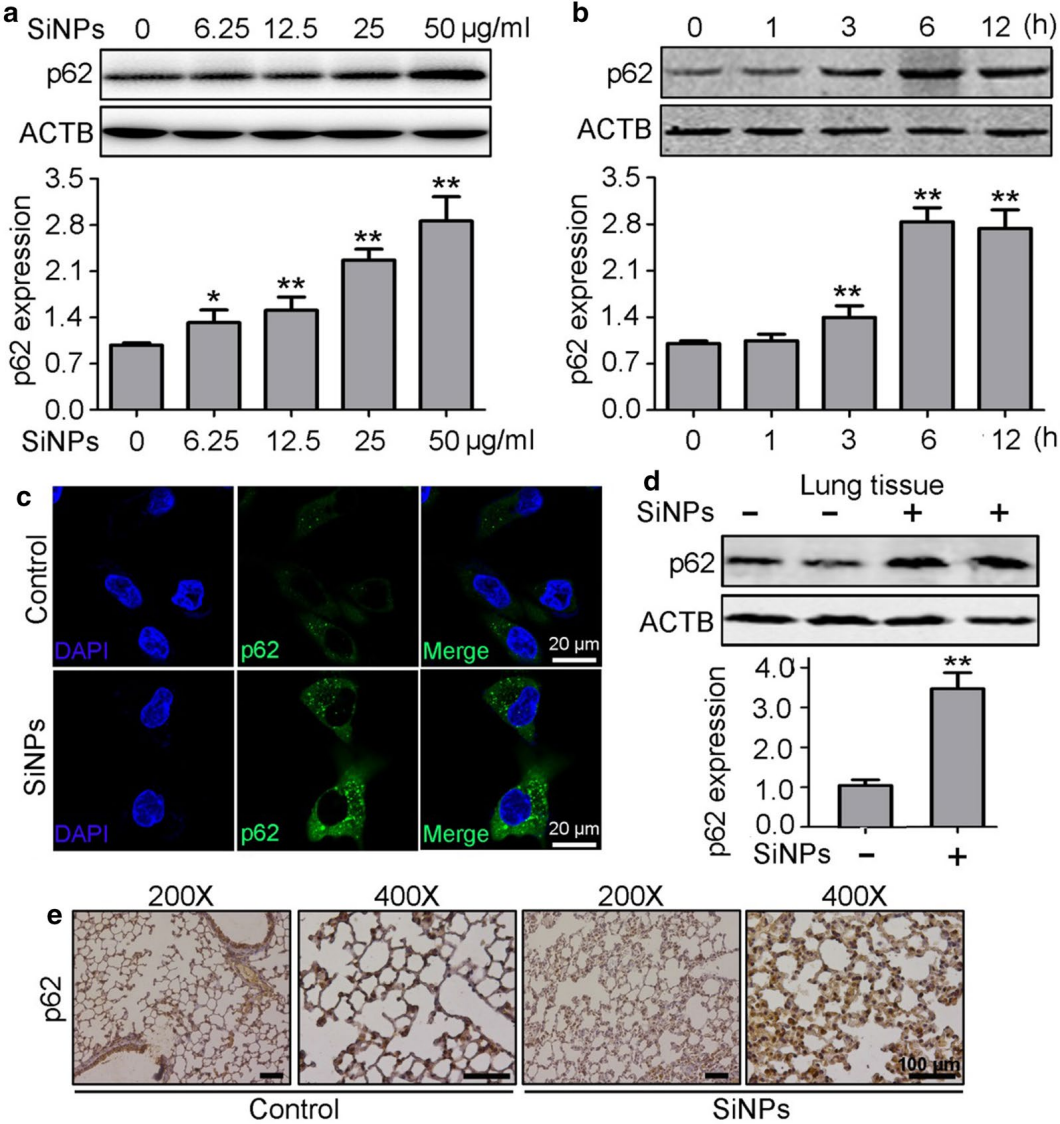
SiNPs induced p62 protein accumulation. **a** BEAS-2b cells were treated with SiNPs at indicated concentrations for 12 h, followed by the cellular p62 protein expression detection by Western blot. Quantification shown below represents the relative p62/ACTB expression levels. **b** BEAS-2b cells were exposed to SiNPs at 50 μg/mL for indicated durations. Then the cellular p62 expression were assessed by Western blot. Quantification shown below represents the relative p62/ACTB expression levels. **c** BEAS-2b cells were exposed to SiNPs at 50 μg/mL for 12 h. The p62 protein accumulation was visualized by indirect immunofluorescence assay. Scale bar: 20 μm. **d** The p62 protein expression level in mice lung tissue was detected by Western blot. n = 5 for each group. Quantification shown below represents the relative p62/ACTB expression levels. **e** The p62 protein expression level in mice lung tissue was detected by IHC. n = 5 for each group. Scale bars: 100 μm. All quantitative data are presented as mean ± SEM, **p < 0.01, *p < 0.05

The physicochemical characterization of SiNPs used in present study have been discribed in our previous studies [5, 20]. To explore whether p62 contributes to SiNP-induced cytotoxicity, we first measured its expression in SiNPs-exposed BEAS-2b cells. The results showed that p62 protein levels increased in a dose-dependent manner with SiNP exposure (Fig. 1a). Then, we performed a time course study using SiNP-treated cells, and found that p62 protein levels were gradually upregulated in a time-dependent manner, showing a significant increase within 3 h (Fig. 1b). Immunofluorescence (IF) staining showed more p62-positive punctuated structures in SiNP-exposed cells, indicating p62 accumulation (Fig. 1c). To further verify that SiNP treatment increases the p62 protein level in vivo, we examined its expression through western blot analysis and found increased p62 accumulation in SiNP-treated mouse lung tissue (Fig. 1d). Finally, immunohistochemical staining confirmed greater p62 protein expression in the SiNP-exposed group (Fig. 1e). These data indicate that p62 protein accumulates under SiNP-exposed conditions in vitro and in vivo.

### SiNPs induced p62 accumulation through autophagic flux inhibition resulting from impairment of lysosomal degradative capacity in vitro

**Fig. 2:**
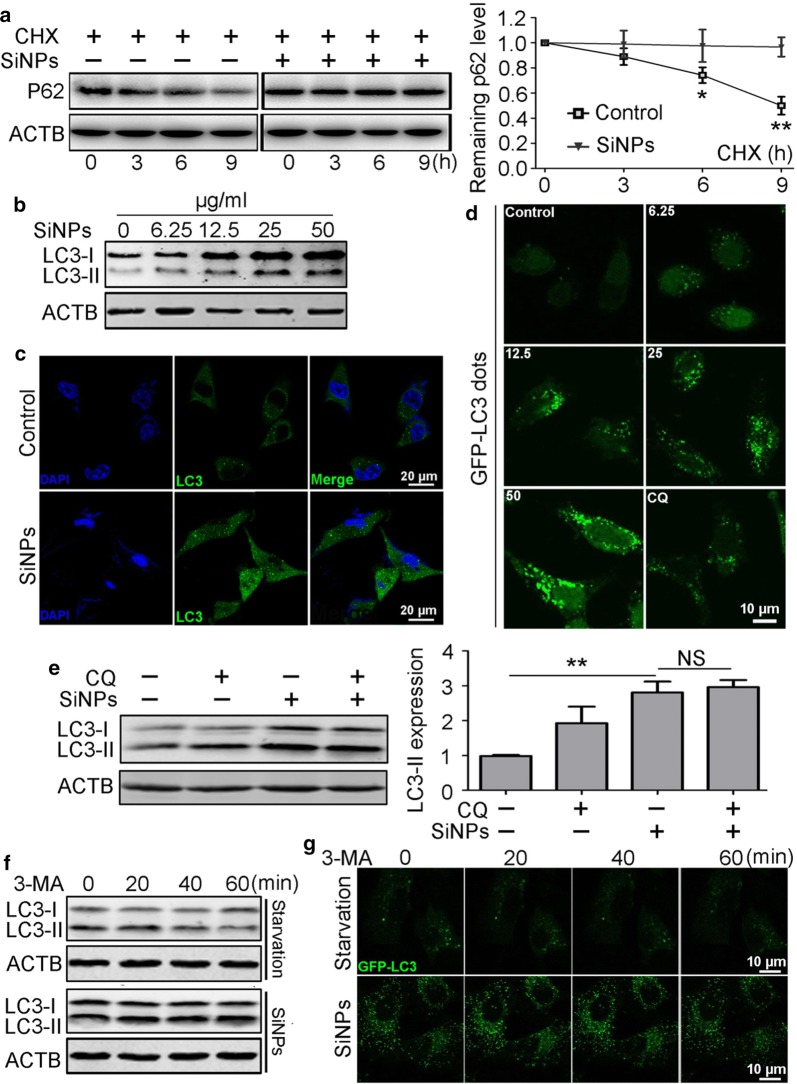
SiNPs inhibited p62 protein degradation through autophagic flux inhibition. **a** The p62 degradation in BEAS-2b cells was detected by Western blot. The graph at right shows the statistical analysis of the remaining p62. **b** BEAS-2b cells were exposed to SiNPs at indicated doses for 12 h, followed by cellular LC3 expression levels measurement by Western blot. **c** BEAS-2b cells were treated with 50 μg/mL SiNPs for 12 h, and the cellular LC3 was evaluated by immunofluorescence. Scale bars: 20 μm. **d** BEAS-2b cells transiently expressing GFP-LC3 were treated with SiNPs at indicated concentrations for 12 h, then were imaged by confocal microscopy. Scale bars: 10 μm. **e** BEAS-2b cells were treated with SiNPs at 50 μg/mL in the absence or presence of CQ (20 μM) for 12 h, and the LC3 protein levels were detected by Western blot. Quantification shown at right illustrates the relative LC3-II/ACTB expression level. **f** Autophagosome degradation in BEAS-2b cells exposed to SiNPs at 50 μg/mL for 12 h or starvation medium were detected by Western blot. **g** BEAS-2b cells transiently expressing GFP-LC3 were treated as in (**f**), and imaged by confocal microscopy. Scale bars: 10 μm. All quantitative data are presented as mean ± SEM, **p < 0.01, *p < 0.05, NS: no significance

Next, we investigated the molecular mechanism underlying SiNP-induced p62 accumulation in vitro. As p62 degradation in lysosomes is important for normal autophagy, we directly measured its degradation through pre-treatment with CHX, a protein synthesis inhibitor. The results showed that p62 was gradually degraded in control cells and that SiNP treatment significantly inhibited its degradation, suggesting possible dysfunction of autophagy (Fig. 2a). We subsequently assessed autophagosome accumulation based on LC3 protein levels, and found that SiNPs increased LC3-II expression levels in a dose-dependent manner in BEAS-2b cells (Fig. 2b). IF staining consistently showed more LC3-positive dots in SiNP-exposed cells (Fig. 2c). Furthermore, more GFP-LC3 punctuated structures were observed in BEAS-2b cells transiently expressing GFP-LC3 (Fig. 2d).

Next, we performed autophagic flux analysis using CQ, a classic lysosome degradation inhibitor. The results showed that CQ treatment failed to further increase LC3-II protein levels in SiNP-exposed cells, indicating blockade of autophagic flux (Fig. 2e). Finally, we inhibited new autophagosome formation by treatment with 3-MA, then detected the number of autophagosomes to assess the autophagosomes degradation. In starved cells, autophagosomes were gradually degraded, whereas their degradation was inhibited in SiNP-treated cells. Moreover, we tracked autophagosome degradation in cells expressing GFP-LC3 and found that GFP-LC3 dots were retained in SiNP-exposed cells after 3-MA treatment, directly confirming autophagosome degradation inhibition. Collectively, our data suggest that SiNPs inhibit autophagic flux, leading to p62 accumulation.

**Fig. 3:**
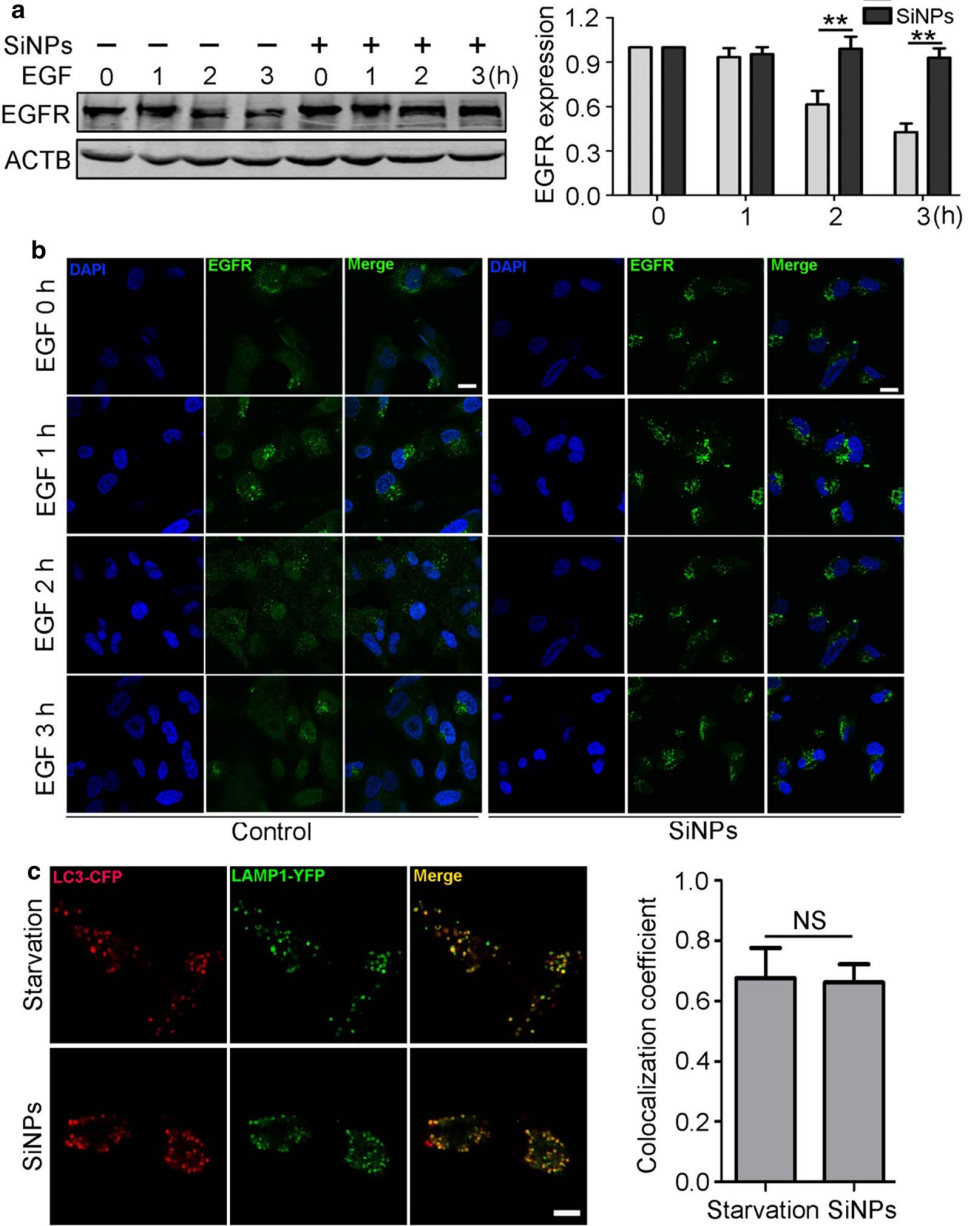
SiNPs impaired lysosomal degradative capacity. **a** The EGFR degradation in BEAS-2b cells treated with 50 μg/mL of SiNPs for 12 h was detected by Western blot. Quantification shown at right illustrates the relative EGFR/ACTB expression level compared to 0 h. **b** BEAS-2b cells were treated as indicated in (**a**), then were fixed and immunostained with anti-EGFR antibody. Scale bars: 10 μm. **c** BEAS-2b cells transiently expressing CFP-LC3 and YFP-LAMP1 were either starved or exposed to SiNPs at 50 μg/mL for 12 h, then the cells were fixed and imaged by confocal microscopy. The graph at right shows a statistical assay of the colocalization coefficients of LC3-CFP and LAMP1-YFP. Scale bars: 10 μm. Data are presented as mean ± SEM, **p < 0.01, *NS* no significance

Because the lysosome is the final destination for autophagosome degradation, we evaluated lysosomal degradation capacity through an EGFR degradation assay. This immunoblotting assay showed that SiNPs retarded cellular EGFR degradation after EGF stimulation (Fig. 3a). Furthermore, IF showed that EGFR dots were largely retained in SiNP-exposed cells. However, a dramatic portion of endocytosed EGFR was degraded in untreated cells (Fig. 3b). The complete autophagic flux process also entails autophagosome fusion with lysosomes prior to cargo degradation. Therefore, we evaluated autophagosome-lysosome fusion by assessing the colocalisation of CFP-LC3 with YFP-LAMP1. The results showed no difference in colocalisation between starved cells and SiNP-treated cells (Fig. 3c). Taken together, these results show that SiNPs inhibited autophagic flux through impairment of lysosomal degradative capacity rather than autophagosome-lysosome fusion.

### SiNPs activated p62 transcription in an Nrf2-dependent manner in vitro

**Fig. 4:**
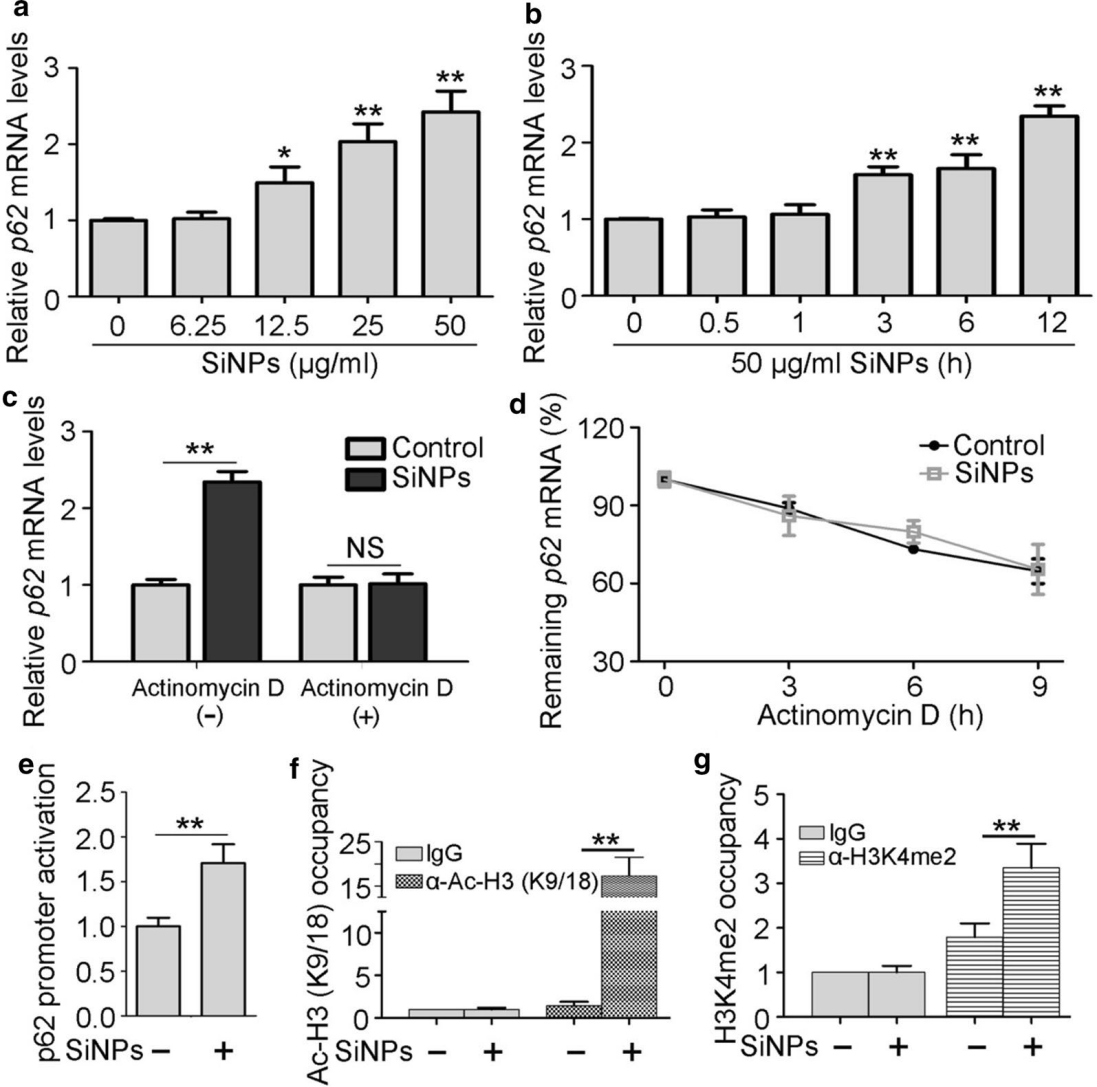
The transcription of p62 gene was activated by SiNPs. **a** BEAS-2b cells were treated with different concentrations of SiNPs for 12 h, and *p62* mRNA levels were determined by qRT-PCR. **b** The cells were treated with SiNPs at 50 μg/mL for indicated durations, and *p62* mRNA levels were determined by qRT-PCR. **c** BEAS-2b cells were exposed to SiNPs at 50 μg/mL for 12 h in the absence or presence of actinomycin D (2 μg/mL), and the *p62* mRNA level was measured by qRT-PCR. **d** The *p62* mRNA degradation in BEAS-2b cells exposed to SiNPs was detected by qRT-PCR. **e** BEAS-2b cells transfected with p62 promoter luciferase reporter and renilla luciferase internal control were treated with or without 50 μg/mL SiNPs for 12 h. Luciferase activity was then detected and normalized to renilla activity. **f**, **g** BEAS-2b cells were treated with or without 50 μg/mL SiNPs for 12 h. ChIP analysis was performed with antibodies against Ac-H3(K9/18) (**f**) or H3K4me2 (**g**), then analyzed by qRT-PCR. All quantitative data are presented as mean ± SEM. **p < 0.01, *p < 0.05, NS: no significance

In response to various stresses, p62 accumulation is also driven by pre-translational regulation [21]; therefore, we measured its mRNA expression levels after SiNP treatment. The results showed that *p62* mRNA expression levels increased dramatically in SiNP-exposed BEAS-2b cells in a dose- and time-dependent manner (Fig. 4a, b). To determine whether the elevated level of *p62* mRNA is caused by transcription activation or post-transcriptional regulation, actinomycin D was applied to block de novo RNA synthesis. De novo RNA synthesis inhibition effectively prevented cells from exhibiting SiNP-stimulated *p62* mRNA upregulation (Fig. 4c), suggesting that p62 is regulated at the transcriptional level. Conversely, *p62* mRNA stability did not change during SiNP treatment, eliminating the possibility of post-transcriptional regulation (Fig. 4d). Moreover, we directly assessed p62 gene promoter activity using a luciferase reporter and found that the transcriptional activity of the p62 promoter region increased dramatically upon SiNP exposure (Fig. 4e).

**Fig. 5:**
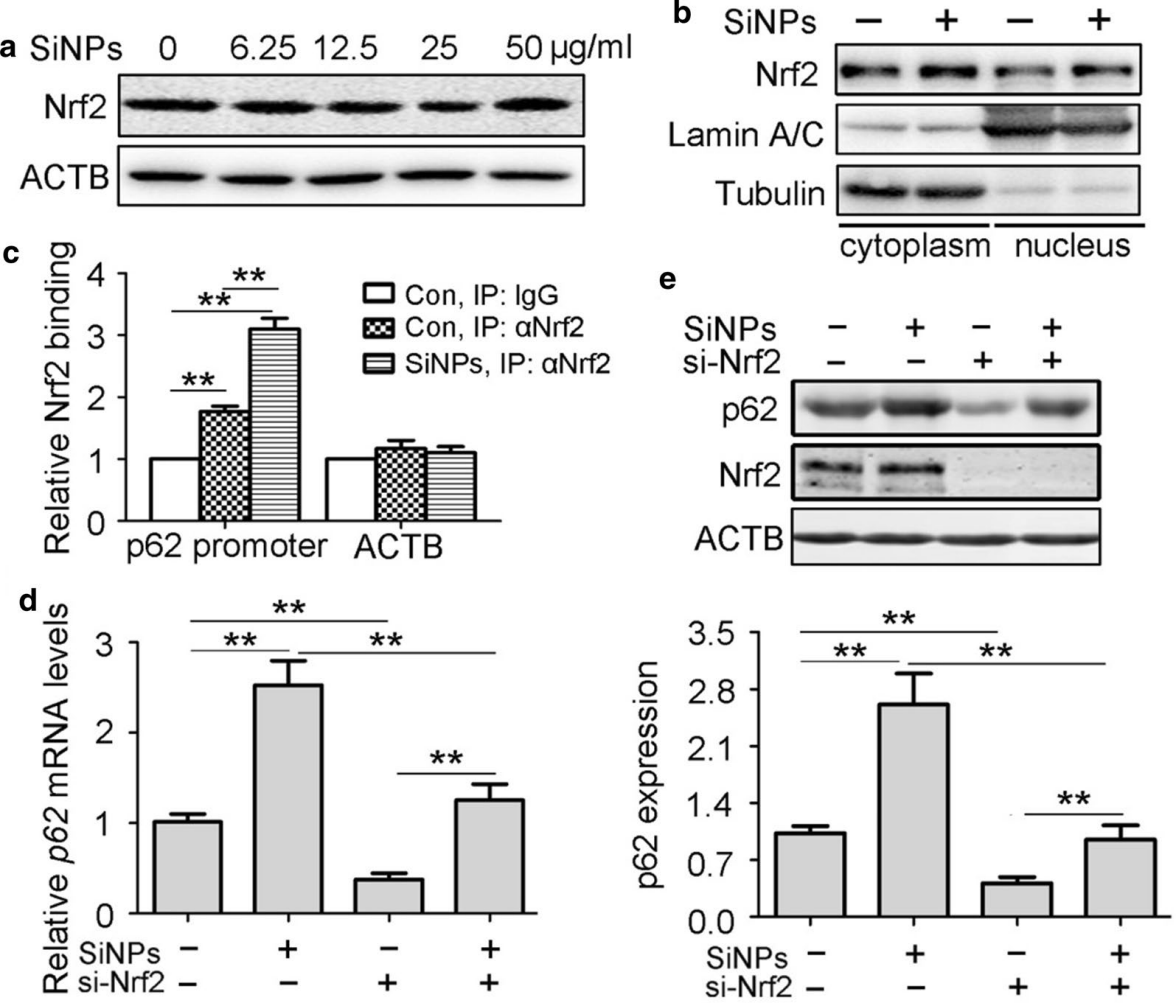
Nrf2 mediated SiNPs-stimulated p62 transcription activation. **a** BEAS-2b cells were treated with SiNPs at indicated doses for 12 h, and Nrf2 protein expression levels were detected by Western blot. Quantification shown below represents the relative Nrf2/ACTB expression level. **b** BEAS-2b cells were treated with SiNPs at 50 μg/mL for 12 h, then Nrf2 expressions in nucleus or cytoplasm were detected by western blot. **c** BEAS-2b cells treated with or without SiNPs at 50 μg/mL for 12 h. ChIP analysis was performed with antibody against Nrf2 and analyzed by qPCR. **d** BEAS-2b cells transfected with siRNA targeting Nrf2 or control sequence were treated with or without SiNPs at 50 μg/mL for 12 h, followed by detection of *p62* mRNA expression using qRT-PCR. **e** BEAS-2b cells were treated as indicated in (**d**), followed by detection of p62 protein expression using western blot. Quantification shown below represents the relative p62/ACTB expression level. All quantitative data are presented as mean ± SEM. **p < 0.01, *p < 0.05

Acetylation modification of histones at gene promoters facilitates efficient polymerase transit by loosening histone-DNA contacts, therefore activating transcription [22]. Histone H3 has been found to be dimethylated at lysine 4 (K4) in active euchromatic regions but not in silent heterochromatic sites [23]. Collectively, Ac-H3(K9/18) and H3K4me2 have been identified as active gene markers [24]. Thus, we measured Ac-H3(K9/18) and H3K4me2 levels at the *p62* promoter region using chromatin immunoprecipitation (ChIP). The results showed that SiNP treatment markedly increased both Ac-H3(K9/18) and H3K4me2 levels at the *p62* promoter region, providing further evidence of transcriptional activation (Fig. 4f, g). Based on the presence of an antioxidant response element (ARE) in the p62 promoter region, which is a consensus Nrf2-binding sequence, we explored whether Nrf2 promoted p62 transcription via binding the ARE [25]. We evaluated the Nrf2 signal pathway after SiNP stimulation. Interestingly, the nuclear localisation of Nrf2 increased after SiNP treatment, but Nrf2 expression was not affected (Fig. 5a, b). Then, the ChIP assay was used to determine the direct binding of Nrf2 to the p62 promoter. The results showed a significant enrichment of the p62 promoter region but not the ACTB gene following immunoprecipitation with the Nrf2 antibody. Moreover, SiNP exposure dramatically stimulated Nrf2 binding to the p62 promoter (Fig. 5c). To further confirm the role of endogenous Nrf2 in p62 expression regulation, siRNA was used (Additional file 1: Fig. S1a, b). Knockdown of Nrf2 led to significant reduction of both p62 mRNA and protein expression under normal conditions as well as with SiNP treatment (Fig. 5d, e). Taken together, these data indicate that Nrf2 binds the p62 promoter and thus mediates its transcription.

### SiNP-associated p62 triggered inflammation through NF-κB activation in vitro

**Fig. 6:**
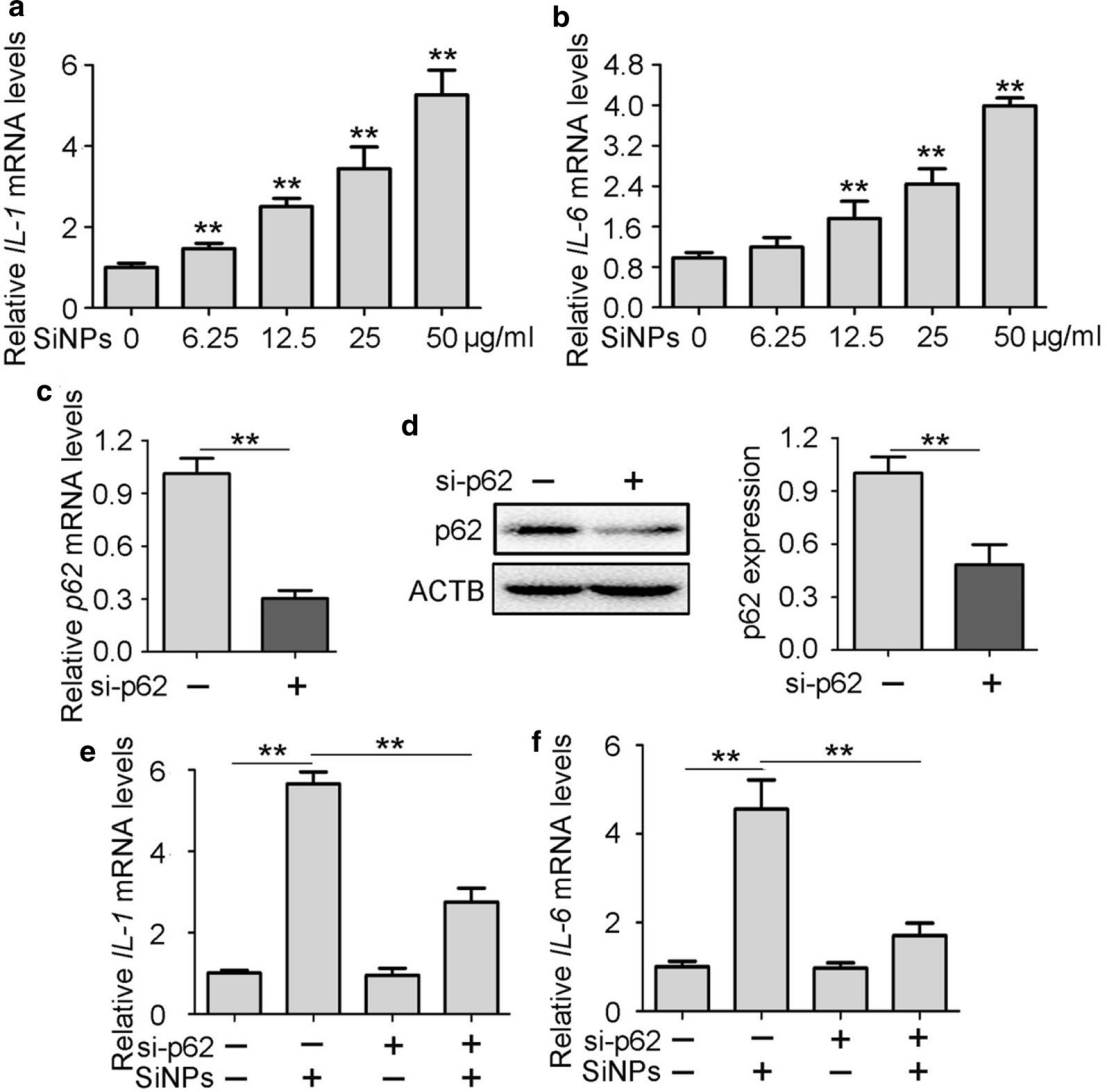
p62 accumulation mediated SiNPs-stimulated inflammation. **a**, **b** BEAS-2b cells were treated with different concentrations of SiNPs for 12 h, then *IL*-*1* (**a**) and *IL*-*6* (**b**) mRNA levels were determined by qRT-PCR. **c**, **d** BEAS-2b cells were transfected with siRNA targeting p62 or control sequence, then *p62* mRNA and protein expressions were detected by qRT-PCR (**c)** and western blot (**d**) respectively. Quantification right shows the relative p62/ACTB expression level. **e**, **f** BEAS-2b cells transfected with siRNA targeting p62 or control sequence were treated with or without SiNPs at 50 μg/mL for 12 h, followed by detection of *IL*-*1* (**e**) and *IL*-*6* (**f**) mRNA expression using qRT-PCR. All quantitative data are presented as mean ± SEM. **p < 0.01

Having established the involvement of autophagy and p62 in regulation of the inflammatory immune response, we examined whether p62 accumulation mediates SiNP-triggered airway inflammation in vitro [26, 27]. After treatment with SiNPs, *IL*-*1* and *IL*-*6* gene expression levels increased dramatically in a dose-dependent manner (Fig. 6a, b). To observe the role of p62 accumulation in SiNP-induced inflammation, knockdown of p62 was performed (Fig. 6c, d). Silencing of p62 using siRNA significantly attenuated SiNP-induced *IL*-*1* and *IL*-*6* expression (Fig. 6e, f). Similarly, p62 knockdown alleviated their release into culture medium. These data suggest that p62 accumulation is critically involved in SiNP-mediated inflammation in vitro.

**Fig. 7:**
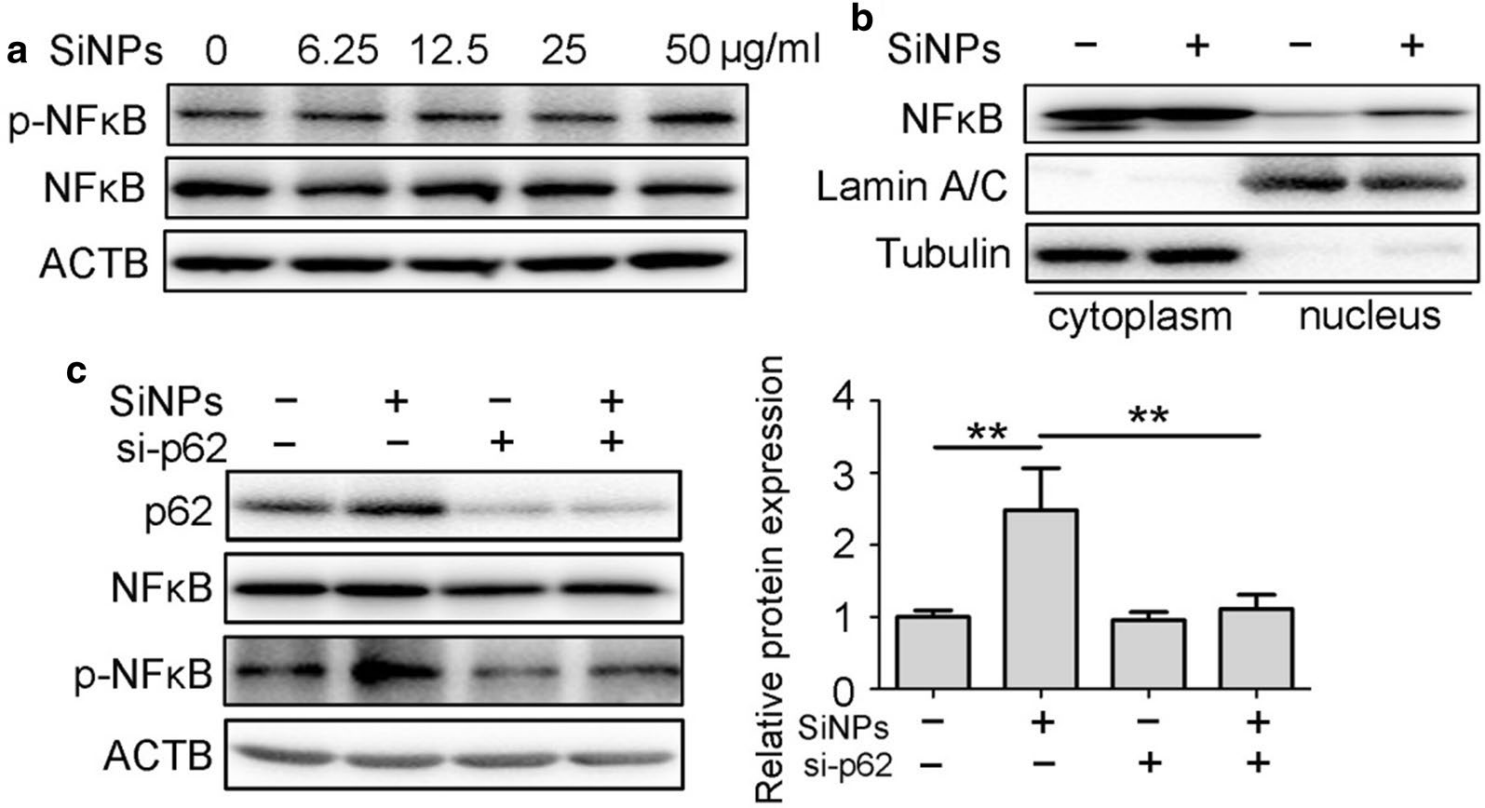
p62 accumulation mediated inflammation through NF-κB activation. **a** BEAS-2b cells were treated with SiNPs at indicated concentrations for 12 h, followed by the cellular NF-κB as well as p-NF-κB protein expression detection by Western blot. **b** BEAS-2b cells were treated with SiNPs at 50 μg/mL for 12 h, then NF-κB expressions in nucleus or cytoplasm were detected by western blot. **c** BEAS-2b cells transfected with siRNA targeting p62 or control sequence were treated with or without SiNPs at 50 μg/mL for 12 h, followed by detection of p62, NF-κB as well as p-NF-κB protein expression. Quantification shown right represents the relative p-NFκB/NFκB expression level. All quantitative data are presented as mean ± SEM. **p < 0.01

Recent studies have reported that the adaptor protein p62 interacts with NF-κB to mediate inflammation [28]. We therefore examined whether SiNP-induced p62 accumulation functioned through NF-κB activation. As expected, treatment with SiNPs induced over-phosphorylation of NF-κB and its translocation to the nucleus from the cytoplasm (Fig. 7a, b). p62 siRNA effectively reduced NF-κB phosphorylation in SiNP-exposed cells, showing the contribution of p62 accumulation to SiNP-stimulated NF-κB activation (Fig. 7c).

## Discussion

**Fig. 8:**
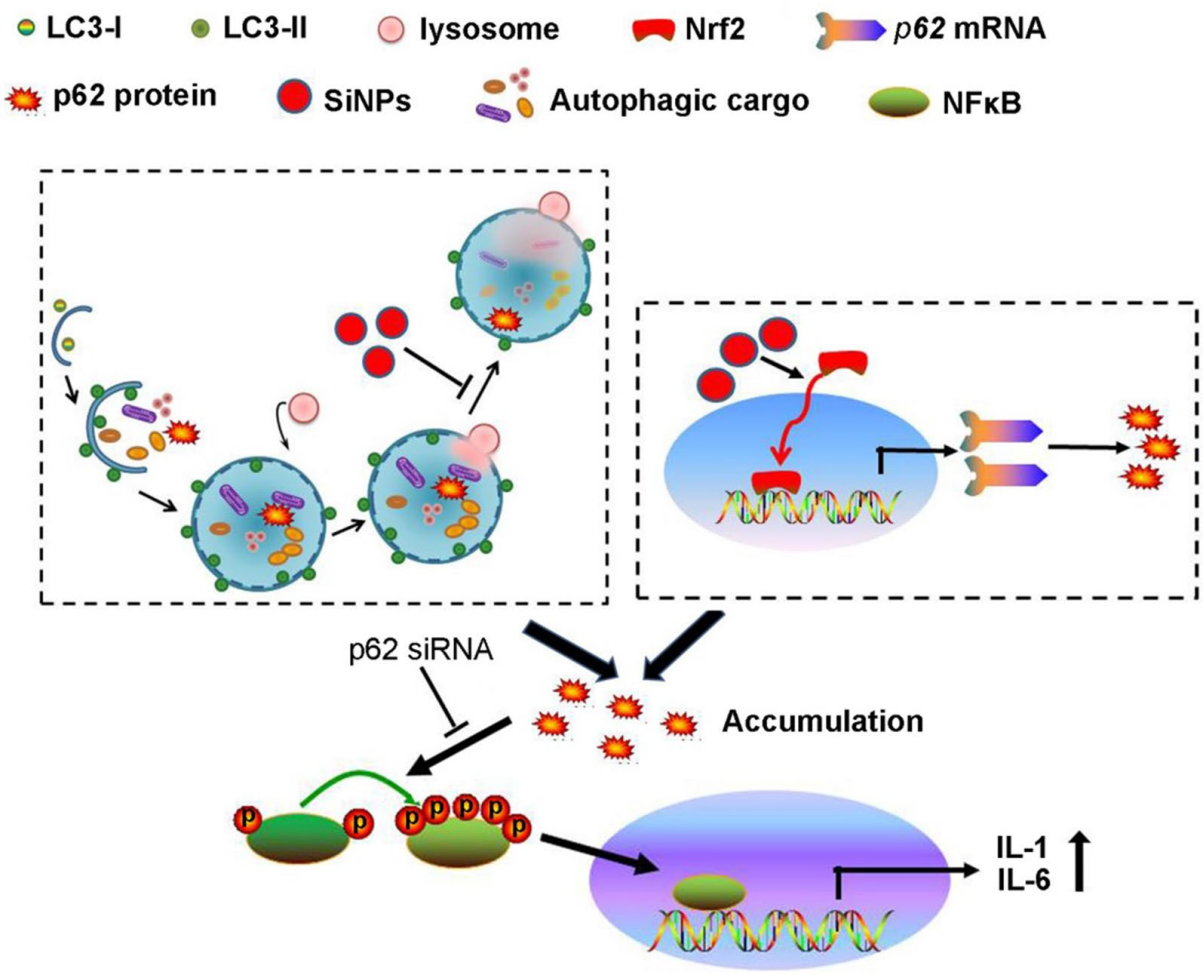
A scheme diagram deciphering the mechanism underlying SiNPs-induced p62 accumulation and subsequent airway inflammation

Although SiNPs trigger airway inflammation, the underlying molecular mechanisms remain largely unclear. This study showed that SiNP led to accumulation of p62 due to both suppression of lysosomal impairment-mediated degradation and Nrf2-dependent transcriptional activation. Once accumulated, p62 activated NF-κB and increased its translocation to the nucleus from the cytoplasm, resulting in increased expression of IL-1 and IL-6 (Fig. 8).

SiNPs triggered pulmonary pro-inflammatory responses both in vivo and *vitro* [29, 30]. NF-κB, a classical inflammatory mediator, has been detected in several studies. However, whether SiNPs activate this response has remained controversial due to differences among experimental models. For example, no significant activation of NF-κB could be detected in SiNP-treated human endothelial cells [31]. By contrast, SiNPs induced a cytokine response in lung epithelial cells through NF-κB signalling [32]. Here, we reported that SiNPs activate NF-κB in a p62 accumulation-dependent manner. The ubiquitin ligase tumour necrosis factor receptor-associated factor 6 (TRAF6) is a key regulator, and its activation and polyubiquitination is critical to NF-κB activation [33]. Moreover, p62 interacts with TFAF6 through its TRAF6 binding domain, facilitating K63-polyubiquitination of TFAF6, and thereby mediating NF-κB activation [12, 34]. A recent report showed that Src-suppressed-C kinase substrate (SSeCKS)-TRAF6 interaction contributed to NF-κB activation induced by exposure to 2,3,7,8-tetrachlorodibenzo-p-dioxin (TCDD) [35]. As SSeCKS is a substrate of protein kinase C, which also directly interacts with p62, we hypothesised that p62 functions as a signalling hub through interactions with multiple binding partners [12, 36]. Further efforts to identify binding proteins of p62 and explore their roles in SiNP-induced NF-κB activation are urgently needed.

In the present study, we assessed the role of p62 accumulation in SiNP-triggered airway inflammation. Once inhaled, SiNPs could stimulate apoptosis, oxidative stress, and the epithelial to mesenchymal transition (EMT) in addition to inflammation [3, 5, 37]. Interestingly, these cellular outcomes, including the EMT, are also mediated by p62 accumulation [38]. NF-κB activation contributed to p62-mediated EMT, thereby promoting nasopharyngeal carcinoma [39]. In particular, Feng et al. reported that GO caused caspase-mediated apoptosis due to aberrant p62 accumulation, suggesting that p62 accumulation is involved in NP-associated cytotoxicity through multiple mechanisms [16]. A recent publication indicated that enhancement of autophagy can attenuate mesoporous silica nanoparticle-induced NF-κB-dependent inflammation, although it lacked assessment of p62 expression [40]. Our results showed that accelerated autophagic flux decreased p62 expression and alleviated SiNP-triggered airway inflammation. To build a direct link between p62 accumulation and inflammation, we downregulated p62 using siRNA and found that p62 knockdown inhibited *IL*-*1* and *IL*-*6* expression (Fig. 6). Zinc ions released from ZnO NPs damaged lysosomes, leading to impairment of autophagic flux and subsequently of mitochondrial accumulation. This process could cause excessive oxidative stress, suggesting that autophagic flux inhibition functions not only through p62 accumulation [41]. Therefore, we should distinguish the role of p62 accumulation from that of autophagic flux blockade using gene methods to explore NP-associated toxicity.

Protein expression can be regulated via pre- or post-translational mechanisms. In previous research, investigators measured p62 expression to assess autophagic flux [42]. Unsurprisingly, SiNPs inhibited p62 degradation through lysosomal impairment. We found that SiNPs were transported from the early endosome to lysosome following caveolae-mediated endocytosis, indicating that the lysosome is the main target of SiNPs [20]. Almost all NPs have been shown to target and damage the lysosome, thereby blocking autophagic flux. For example, silver NPs inhibited autophagic flux through lysosomal pH alkalisation [43]. SiNPs induced autophagy dysfunction via lysosomal impairment in hepatocytes [44]. All of these processes were accompanied by p62 accumulation. Thus, autophagic flux blockade-mediated p62 accumulation may be a common toxicological mechanism upon NP exposure, as p62 regulates multiple cellular outcomes. To our knowledge, no studies to date have analysed p62 regulation by NPs at the pre-translational level. This is the first report showing that NPs can enhance p62 transcriptional activation in an Nrf2-dependent manner, further supporting the occurrence of multiple p62-regulated processes upon NP exposure. Gene expression programs depend on the recognition of specific sequences by transcriptional regulatory proteins. Here, we determined that Nrf2 could bind to the p62 gene promoter and regulate its expression. Most, if not all, genes are controlled by a combination of transcription factors rather than a single factor [45]. The transcription factor specificity protein 1 (Sp1) enhanced the expression levels of p62 by directly binding to the p62 promoter [46]. MicroRNAs, another large family of gene regulatory molecules found in eukaryotic organisms, are also regulated by SiNPs [47]. Overall, the molecular details of NP-mediated p62 transcription regulation are complex and need further investigations.

## Conclusions

In summary, we provide the first assessment of the role of p62 in SiNP-induced damage. We found that p62 accumulation results from both lysosomal impairment and Nrf2-mediated transcription activation, and subsequently triggers NF-κB activation and inflammatory response. These findings propose a common mechanism for NPs-induced autophagic flux blockade and its cellular outcomes.

## Supplementary information

**Additional file 1.** Additional table and figure.

## Data Availability

All data generated or analyzed during this study are included in this published article (and its additional file).
