# Supplementary material for: p62/SQSTM1 accumulation due to degradation inhibition and transcriptional activation plays a critical role in silica nanoparticle-induced airway inflammation via NF-κB activation
Source: J Nanobiotechnology. 2020 May 19;18:77. doi: 10.1186/s12951-020-00634-1 (PMC7236097; doi:10.1186/s12951-020-00634-1)
Supplement: Supplementary file 1 — Additional file 1. Additional table and figure. [file 12951_2020_634_MOESM1_ESM.docx]

**Additional file**

p62/SQSTM1 accumulation due to degradation inhibition and transcriptional activation plays a critical role in silica nanoparticle-induced airway inflammation via NF-κB activation

**Additional Figures**


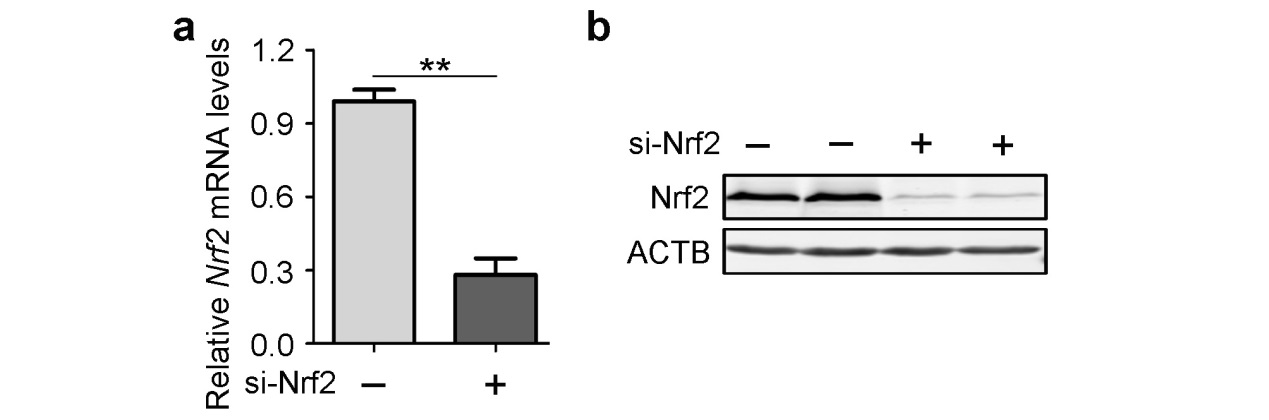


**Figure S1. siRNA effectively downregulated Nrf2 expression.** (a) BEAS-2b cells were transfected with siRNA targerting Nrf2 or control sequence, then *Nrf2* mRNA expressions were detected by qRT-PCR. (b) BEAS-2b cells were transfected with siRNA targerting Nrf2 or control sequence, then Nrf2 protein expressions were evaluated using western blot. ***p*<0.01.

**Additional Tables**

**Table S1.** DNA sequences of primers for polymerase chain reaction.

| **Gene name** | **Primer name** | **Sequence** |
| --- | --- | --- |
| Human *β-actin* | H-ACTB-F | 5’-CACGATGGAGGGGCCGGACTCATC-3’ |
|  | H-ACTB-R | 5’-TAAAGACCTCTATGCCAACACAGT-3’ |
| Human *p62* | H-p62-F | 5’- ATACGGGTGGGAATGTTGAG -3’ |
|  | H-p62-R | 5’- TTCTGGCATCTGTAGGGACTG -3’ |
| Human *IL-1β* | H- *IL-1β*-F | 5’- TGAACTGAAAGCTCTCCACCT -3’ |
|  | H- *IL-1β*-R | 5’- ACTGGGCAGACTCAAATTCCA -3’ |
| Human *IL-6* | H- *IL-6*-F | 5’- TGAGGAGACTTGCCTGGTGA -3’ |
|  | H- *IL-6*-R | 5’- TGCAGGAACTGGATCAGGAC -3’ |
| Human *Nrf2* | H- *Nrf2*-F | 5’- TGCCCCTGGAAGTGTCAAAC -3’ |
|  | H- *Nrf2*-R | 5’- CCCCTGAGATGGTGACAAGG -3’ |
| Human p62 promoter | forward 1 | 5’- TGAGGAGACTTGCCTGGTGA -3’ |
|  | reverse 1 | 5’- TGCAGGAACTGGATCAGGAC -3’ |
| Human p62 promoter | forward 2 | 5’- TGCCCCTGGAAGTGTCAAAC -3’ |
|  | reverse 2 | 5’- CCCCTGAGATGGTGACAAGG -3’ |
